# Supplementary material for: Characteristics of Occupational Exposure to Diesel Engine Exhaust for Shipyard Transporter Signal Workers
Source: Int J Environ Res Public Health. 2020 Jun 18;17(12):4398. doi: 10.3390/ijerph17124398 (PMC7344567; doi:10.3390/ijerph17124398)
Supplement: Supplementary file 1 [file ijerph-17-04398-s001.pdf]

International Journal of
Environmental Research and Public Health

MDPI

Table 1. Daily average wind speed data of research worksite by Beaufort wind scale in 2019.

Class		Wind speed, m/s	Number of dates	Percentage of dates (%)	AM, m/s	Range, m/s
0	Calm	0–0.2	-	-	-	-
1	Light air	0.3–1.5	84	23.0	1.25	0.80–1.50
2	Light breeze	1.6–3.3	206	56.4	2.16	1.50–3.30
3	Gentle breeze	3.4–5.4	38	10.4	4.23	3.31–5.36
4	Moderate breeze	5.5–7.9	27	7.4	6.59	5.59–7.72
5	Fresh breeze	8.0–10.7	8	2.2	9.15	8.06–10.65
6	Strong breeze	10.8–13.8	2	0.5	12.04	11.56–12.53
Total			365	100	2.70	0.80–12.53

Table S2. Correlation coefficients among EC, OC and TC by EC exposure level

	EC < 10 ㎍/㎥ (n=49)			EC≥10 ㎍/㎥ (n=11)			EC data all (n=60)		
	EC	OC	TC	EC	OC	TC	EC	OC	TC
EC	1			1			1		
OC	0.199	1		0.874**	1		0.582**	1	
TC	0.446**	0.961**	1	0.965**	0.971**	1	0.800**	0.948**	1

** P<0.01 correlation is significant at the 0.01 level.
